# Supplementary figures and images for: Mining of co-expression genes in response to cold stress at maize (Zea mays L.) germination and sprouting stages by weighted gene co-expression networks analysis
Source: PeerJ. 2025 Mar 11;13:e19124. doi: 10.7717/peerj.19124 (PMC11908442; doi:10.7717/peerj.19124)

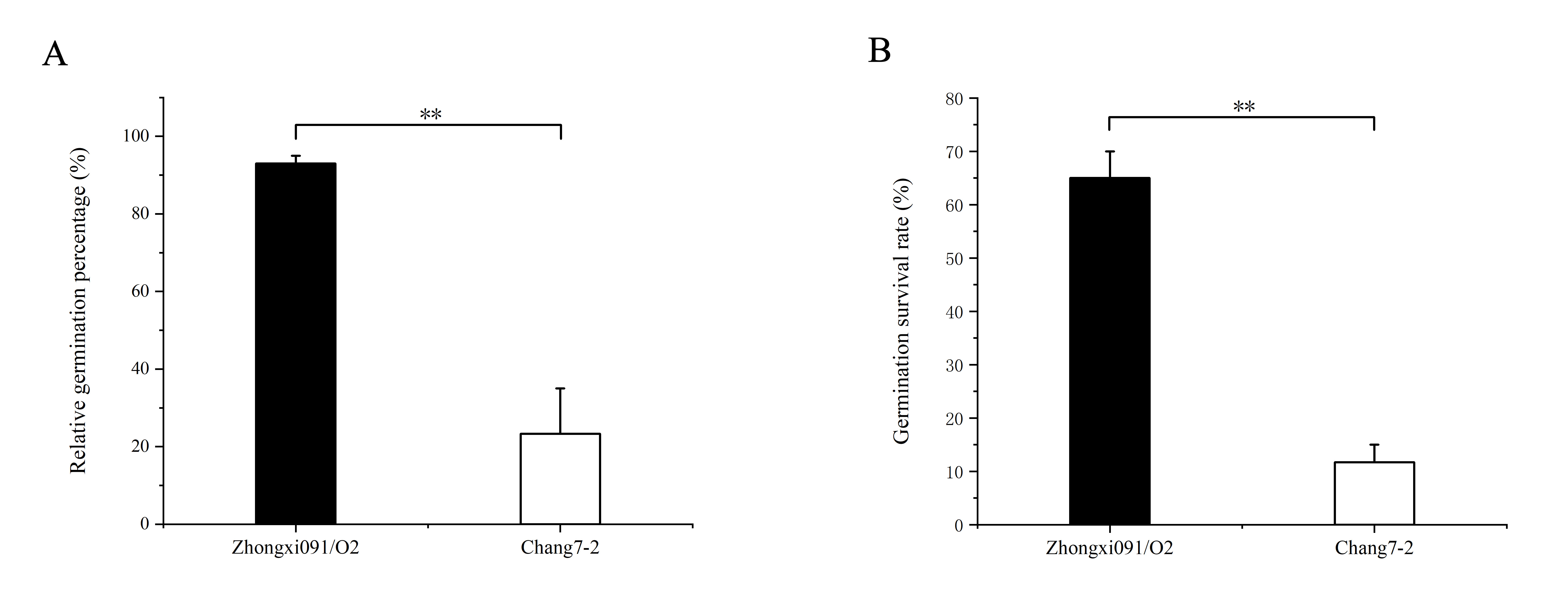

Supplement: Supplemental Information 1 — (A) Relative germination percentage. (B) Germination survival rate. [file peerj-13-19124-s001.jpg]

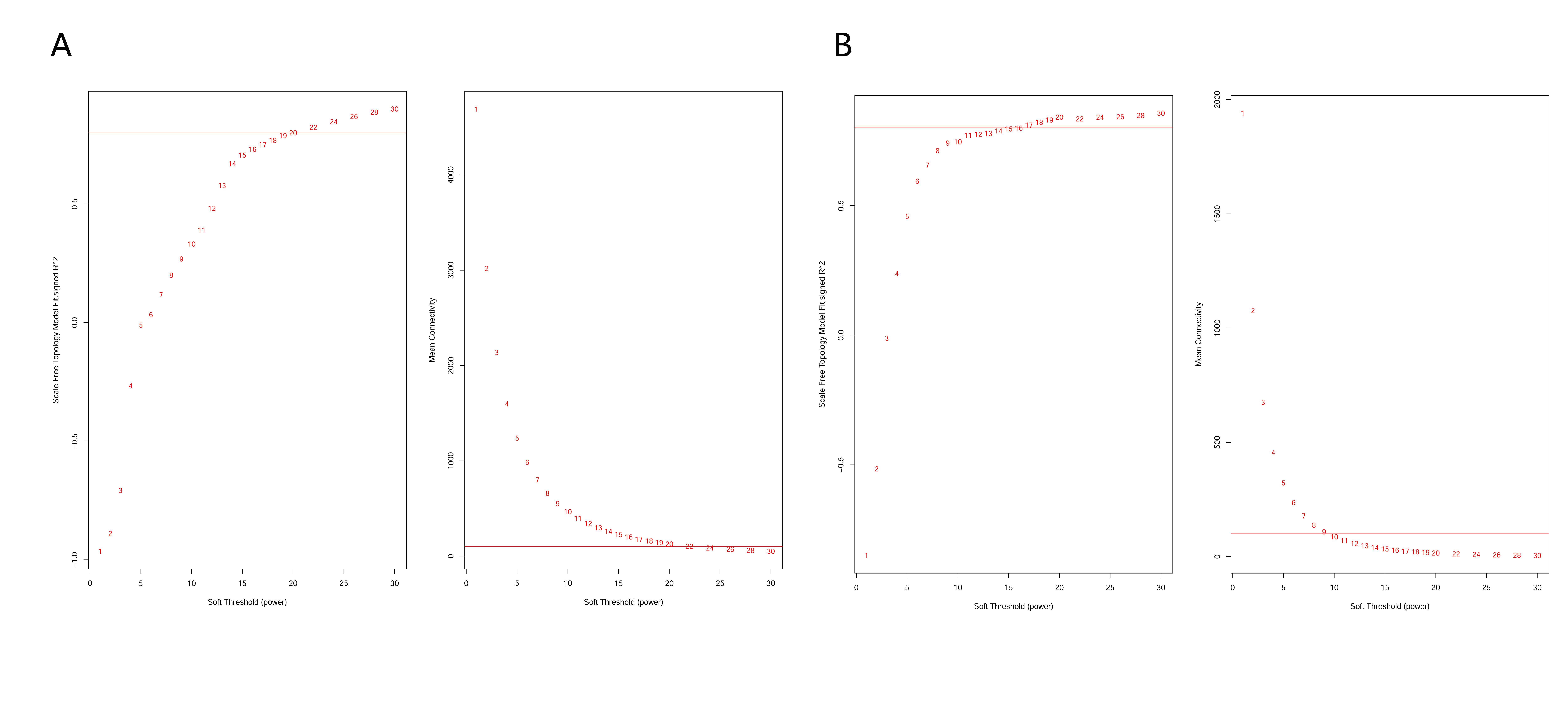

Supplement: Supplemental Information 2 — (A) Germination (G) stage. (B) Sprouting (S) stage. [file peerj-13-19124-s002.jpg]

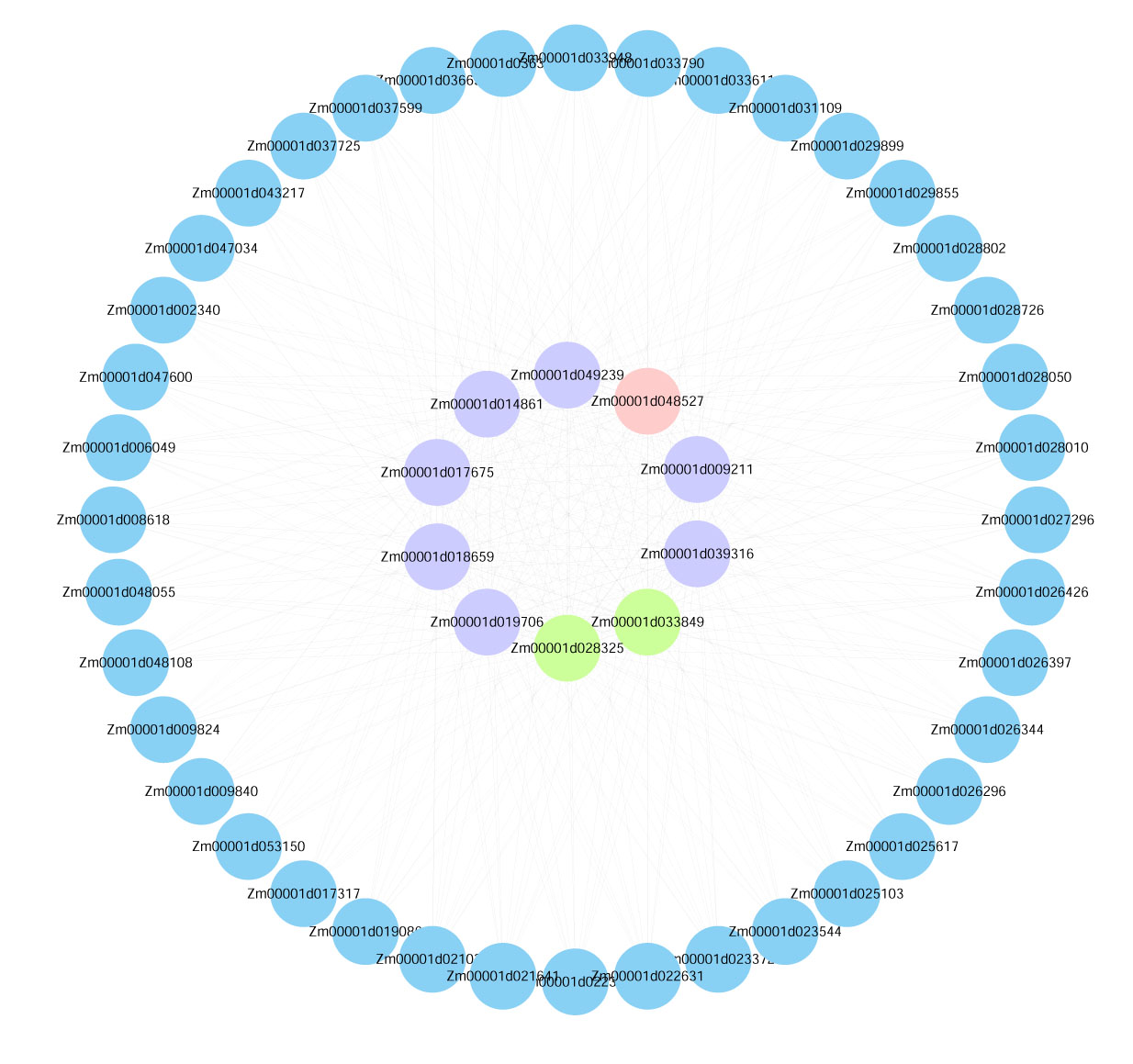

Supplement: Supplemental Information 3 — Note: (Blue Bubble: The top 50 genes in module connectivity. Purple Bubble: The top 10 genes in module connectivity. Green Bubble: The genes proven to be involved in low-temperature stress among the top 10 genes in module connectivity. Pink Bubble: Transcription factors among the top 10 genes in module connectivity.) [file peerj-13-19124-s003.jpg]

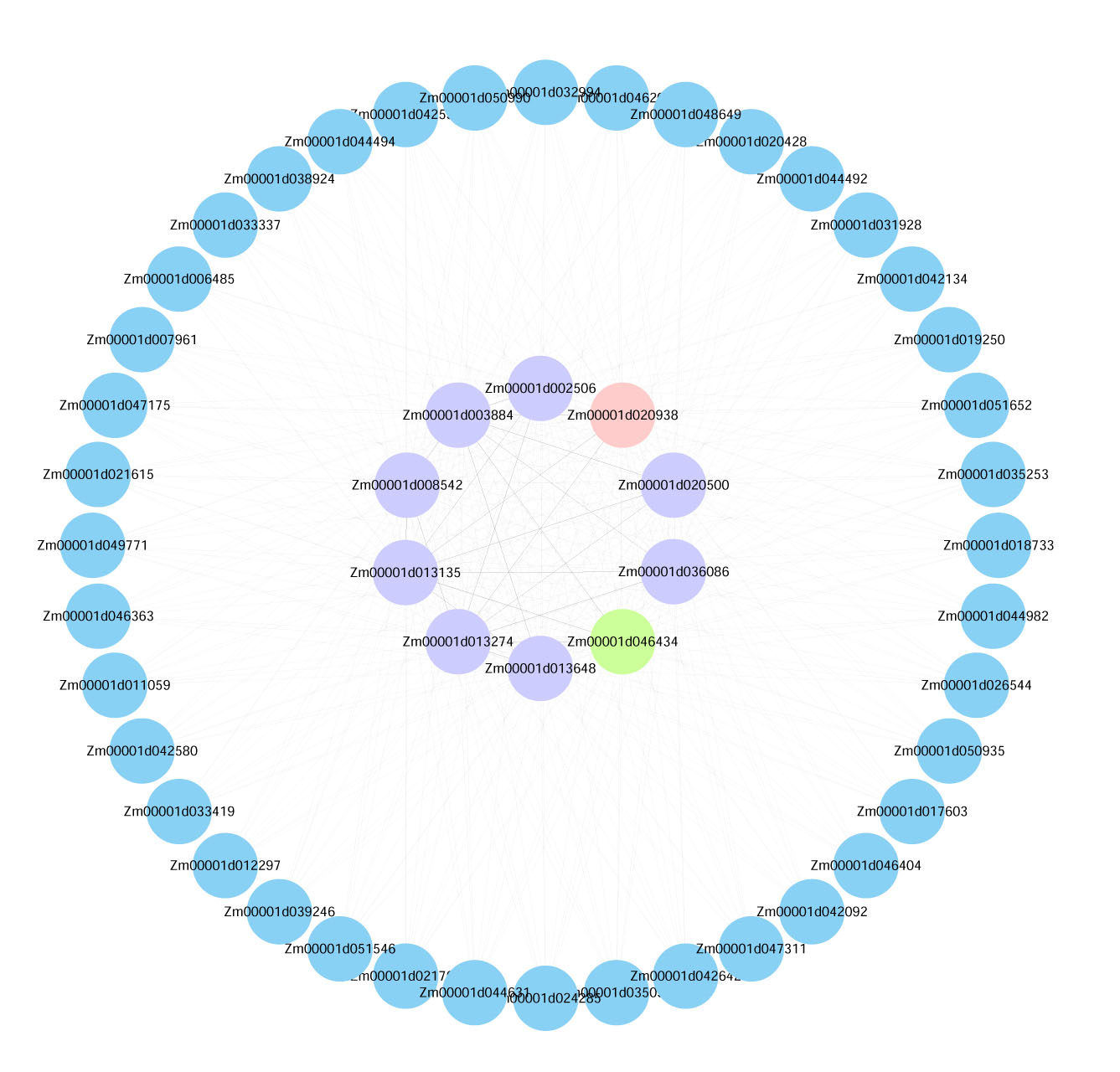

Supplement: Supplemental Information 4 — Note: (Blue Bubble: The top 50 genes in module connectivity. Purple Bubble: The top 10 genes in module connectivity. Green Bubble: The genes proven to be involved in low-temperature stress among the top 10 genes in module connectivity. Pink Bubble: Transcription factors among the top 10 genes in module connectivity.) [file peerj-13-19124-s004.jpg]

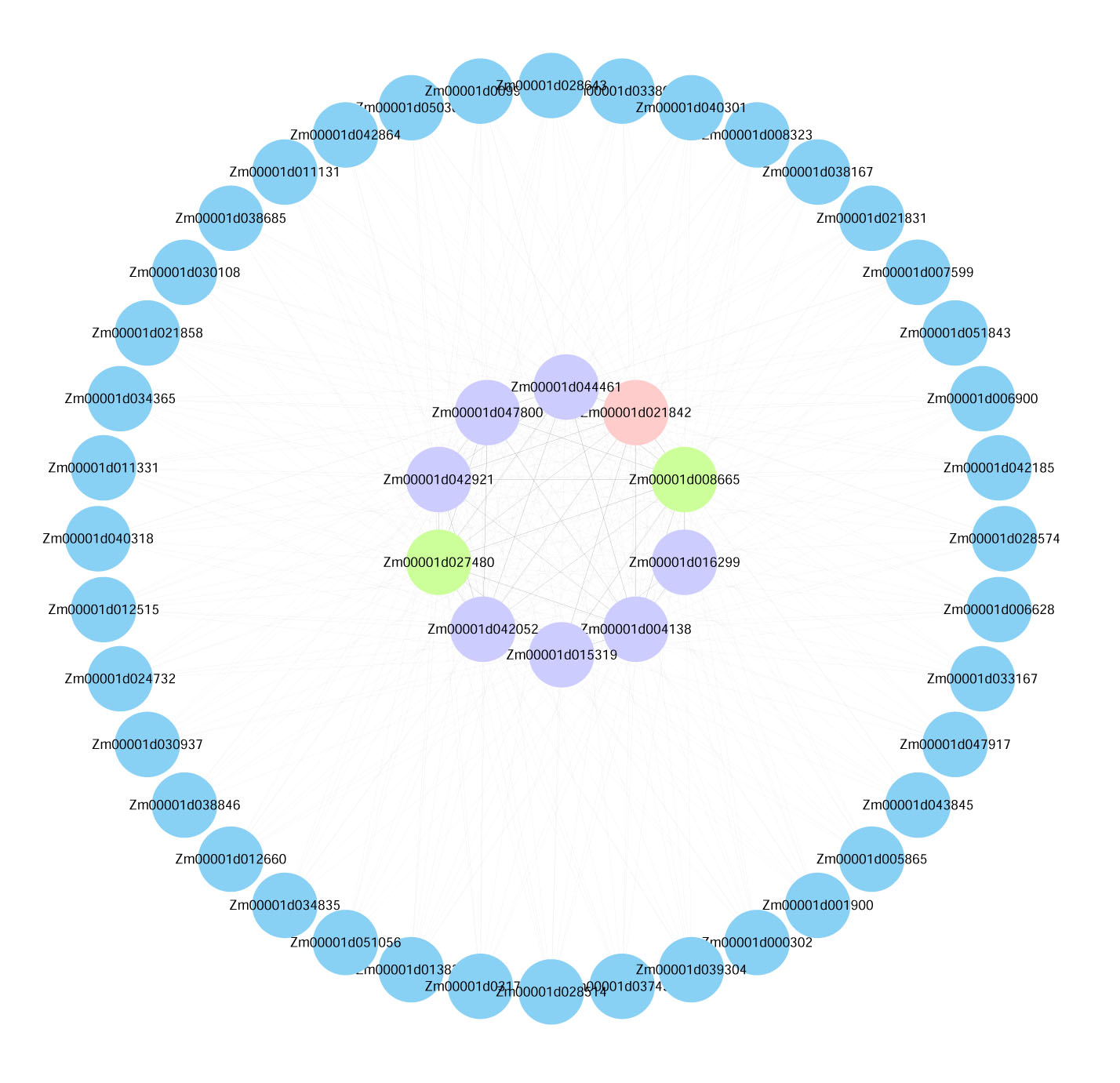

Supplement: Supplemental Information 5 — Note: (Blue Bubble: The top 50 genes in module connectivity. Purple Bubble: The top 10 genes in module connectivity. Green Bubble: The genes proven to be involved in low-temperature stress among the top 10 genes in module connectivity. Pink Bubble: Transcription factors among the top 10 genes in module connectivity.) [file peerj-13-19124-s005.jpg]

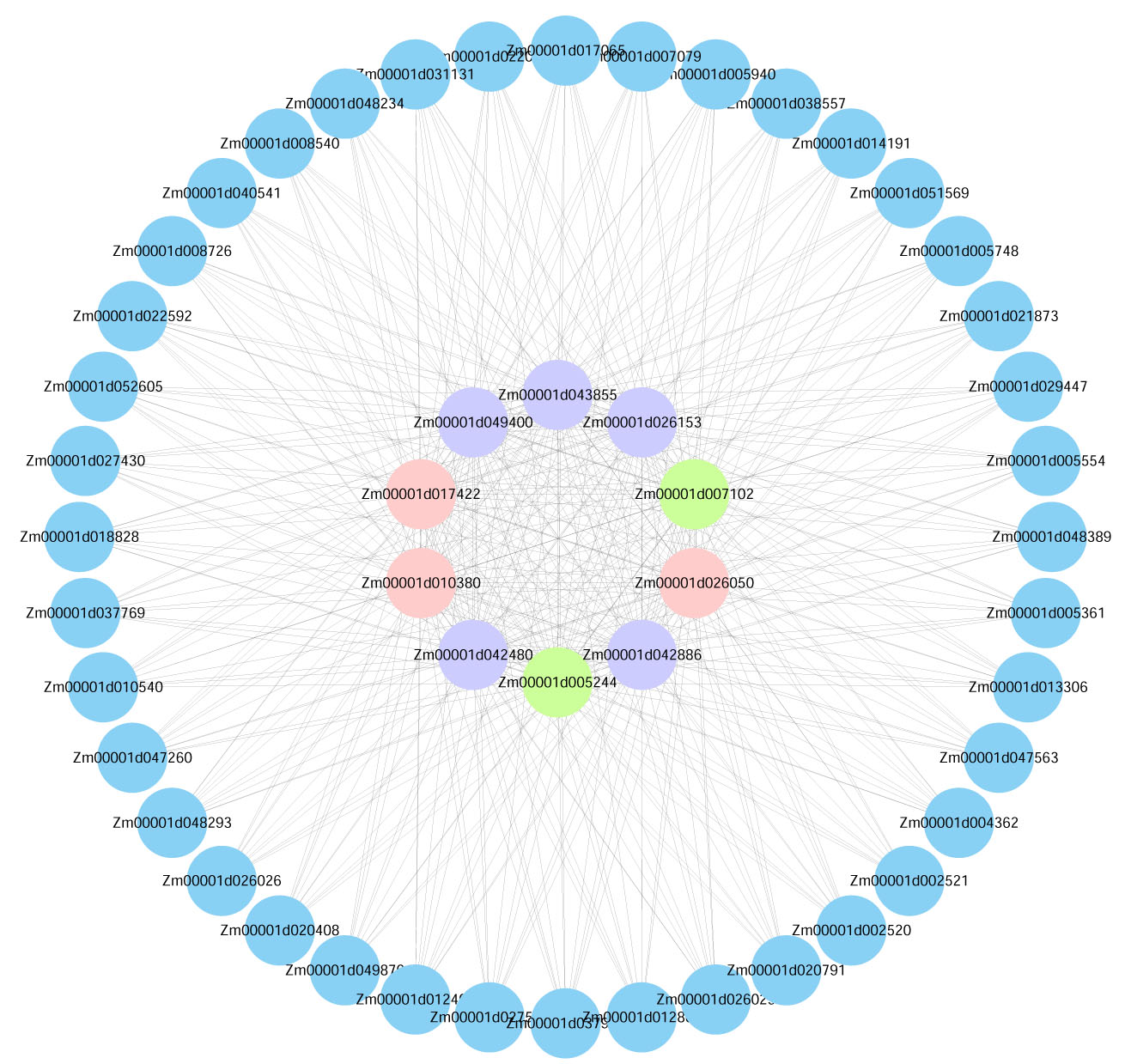

Supplement: Supplemental Information 6 — Note: (Blue Bubble: The top 50 genes in module connectivity. Purple Bubble: The top 10 genes in module connectivity. Green Bubble: The genes proven to be involved in low-temperature stress among the top 10 genes in module connectivity. Pink Bubble: Transcription factors among the top 10 genes in module connectivity.) [file peerj-13-19124-s006.jpg]
